# The Effect of Mindfulness Meditation on Impulsivity and its Neurobiological Correlates in Healthy Adults

**DOI:** 10.1038/s41598-019-47662-y

**Published:** 2019-08-19

**Authors:** Cole Korponay, Daniela Dentico, Tammi R. A. Kral, Martina Ly, Ayla Kruis, Kaley Davis, Robin Goldman, Antoine Lutz, Richard J. Davidson

**Affiliations:** 1 Department of Psychiatry, University of Wisconsin-Madison, Madison, Wisconsin 53719 USA; 2 Department of Psychology, University of Wisconsin–Madison, Madison, Wisconsin 53706 USA; 3 Lyon Neuroscience Research Center, Brain Dynamics and Cognition Team, INSERM U1028, CNRS UMR5292, Lyon 1 University, Lyon, France; 4 Center for Healthy Minds, University of Wisconsin–Madison, Madison, Wisconsin 53703 USA; 5 Waisman Laboratory for Brain Imaging and Behavior, University of Wisconsin–Madison, Madison, Wisconsin 53705 USA; 6 University of Amsterdam, 1012 WX Amsterdam, Netherlands

**Keywords:** Decision, Human behaviour

## Abstract

Interest has grown in using mindfulness meditation to treat conditions featuring excessive impulsivity. However, while prior studies find that mindfulness practice can improve attention, it remains unclear whether it improves other cognitive faculties whose deficiency can contribute to impulsivity. Here, an eight-week mindfulness intervention did not reduce impulsivity on the go/no-go task or Barratt Impulsiveness Scale (BIS-11), nor produce changes in neural correlates of impulsivity (i.e. frontostriatal gray matter, functional connectivity, and dopamine levels) compared to active or wait-list control groups. Separately, long-term meditators (LTMs) did not perform differently than meditation-naïve participants (MNPs) on the go/no-go task. However, LTMs self-reported lower attentional impulsivity, but higher motor and non-planning impulsivity on the BIS-11 than MNPs. LTMs had less striatal gray matter, greater cortico-striatal-thalamic functional connectivity, and lower spontaneous eye-blink rate (a physiological dopamine indicator) than MNPs. LTM total lifetime practice hours (TLPH) did not significantly relate to impulsivity or neurobiological metrics. Findings suggest that neither short- nor long-term mindfulness practice may be effective for redressing impulsive behavior derived from inhibitory motor control or planning capacity deficits in healthy adults. Given the absence of TLPH relationships to impulsivity or neurobiological metrics, differences between LTMs and MNPs may be attributable to pre-existing differences.

## Introduction

The past few decades have seen a surge of interest in the effects of mindfulness meditation on the brain and cognitive functioning. A common aim of various styles of mindfulness meditation is the adoption of a nonreactive and observant stance toward one’s emotions, thoughts and body states, as well as the self-regulation of attention^1^. Thus, on a conceptual basis, mindfulness meditation may be thought to confer benefits for, among other things, improving behavioral control and reducing impulsivity. Impulsivity is a multidimensional construct that may arise from any number of related but distinct cognitive deficits, such as an inability to sustain attention, inhibit prepotent urges, or wait and plan behavior^2^. A number of studies have found that levels of self-reported trait mindfulness are inversely correlated with levels of self-reported trait impulsivity^3–5^.

In light of this conceptual appeal, interest has grown in the prospect of using mindfulness meditation to help treat conditions that feature high levels of impulsivity and deficits in behavioral control, such as attention-deficit/hyperactivity disorder (ADHD)^6^ and substance use disorder (SUD)^7^. While disorders such as ADHD and SUD are multifaceted and likely stem from dysfunction of multiple neurobiological and cognitive domains, treatments that target the impulsive symptoms present in these disorders offer one approach for improving outcomes. In addition, mindfulness meditation may offer a potential strategy for otherwise healthy individuals with high levels of impulsivity to improve their functioning, as high levels of impulsivity in the general population have been linked to poorer life outcomes such as lower levels of academic success^8^ and increased propensity for substance abuse^9^.

However, evidence that mindfulness meditation is effective in reducing impulsivity is sparse. While a number of studies in both healthy individuals^10,11^ and individuals with ADHD^6^ have found evidence that mindfulness meditation can improve attention and reduce attentional impulsivity, it is unclear whether mindfulness meditation is effective in improving other cognitive faculties, such as motor inhibition and long-term planning, whose deficiency can contribute to motor and non-planning impulsivity, respectively. These other dimensions of impulsivity may play a larger role in disorders like SUD^2^, and findings from a recent meta-analysis of 25 studies that found inconclusive data for mindfulness meditation as a treatment for SUD further prompt a more thorough examination of the effect of mindfulness meditation on impulsivity^7^. Only a handful of studies have directly examined the impact of mindfulness meditation interventions on non-attentional measures of impulsivity in healthy adults. One study in healthy adults found that three months of intensive mindfulness meditation training increased subjects’ capacity to inhibit prepotent motor responses on a response inhibition task^12^. Another study found that while mindfulness-based cognitive therapy was effective in improving the ability to inhibit cognitive prepotent responses on the Hayling task, it did not improve the ability to inhibit motor behavior on a GoStop task^13^.

A further issue that has yet to be comprehensively investigated is whether mindfulness meditation has an effect on the neurobiological correlates of impulsivity. Human neuroimaging studies have generally found that impulsivity in healthy adults is negatively correlated with prefrontal gray matter^14–17^ (but see^18,19^). More specifically, several studies have found that self-reported trait measures of impulsivity, such as score on the Barratt Impulsiveness Scale (BIS-11), are negatively correlated with gray matter levels in the anterior cingulate cortex, orbitofrontal cortex, and dorsolateral prefrontal cortex^14–17^ (but see^18,19^). Regarding task-based measures of impulsivity, no study to date has reported a significant relationship between motor inhibition task performance (e.g. on the stop-signal or go/no-go tasks) and gray matter in healthy adults. Studies of impulsivity’s relation to resting-state functional connectivity in healthy adults are sparse and have not yet converged on a consistent set of findings^17,20–23^, but have tended to implicate circuits involving the prefrontal cortical-striatal-thalamic loop, as well as the midbrain. Variation in impulsivity in healthy adults has also been correlated with the catecholaminergic neurotransmitter systems, including the dopaminergic system. Positron emission tomography (PET) studies have found correlations between impulsivity and striatal D2/D3 dopamine receptor availability, though reports diverge on the direction of this relationship^24–26^. Pharmacological challenge studies that administer stimulants such as d-amphetamine have shown that the resulting upregulation of catecholamine levels is associated with reduced motor impulsivity on the go/no-go and stop-signal tasks as well as reduced delay discounting in healthy adults^27^.

A parallel line of literature has also investigated impulsivity’s relation to dopamine using spontaneous eye-blink rate (sEBR), a physiological indicator that convergent evidence suggests may reflect dopaminergic functioning^28–38^. sEBR is thought to vary directly with dopaminergic functioning. In both animals and healthy humans, dopamine agonists increase sEBR while dopamine antagonists decrease sEBR^29–32,34^; in patient cohorts with low dopaminergic functioning^28^, such as those with Parkinson’s disease, sEBR is found to be lower than in healthy individuals. Yet, while findings on the direction of the relationship between sEBR and dopaminergic functioning are generally consistent, findings on the relation of sEBR to impulsivity have varied. Higher sEBR has been associated with poorer inhibitory control on a stop-signal task^39^, and contrarily, with better inhibitory control on the go/no-go task and with lower motor impulsivity as measured by the BIS-11^17^. A further study found that subjects with low sEBR and high self-ratings of disinhibition displayed greater delay discounting^40^. Thus, congruent with its putative underlying regulator (dopamine), sEBR may not have a context-independent, directionally specific relationship with impulsivity, but appears to be meaningfully associated with impulsivity nonetheless.

Previous neuroimaging investigations of the effect of mindfulness meditation on the brain have tended to focus on regions and networks implicated in theory of mind, emotion regulation and attention, with less focus given to effects on the reward and decision-making circuitry detailed above that is relevant to impulsivity. Thus, as of yet it is unclear whether mindfulness meditation causes changes in gray matter, functional connectivity, or dopaminergic functioning in this circuitry.

As such, the present study had two goals. The first was to investigate whether mindfulness meditation training reduces one or more dimensions of impulsivity. The second was to examine whether mindfulness meditation training has an effect on the neural correlates of impulsivity. Given that one aim of mindfulness is to cultivate a state of mind that is nonreactive and observant, and that several studies find self-reported mindfulness and impulsivity are inversely correlated, we hypothesized that a short-term mindfulness intervention could reduce impulsivity in healthy adults, and that long-term meditators would have lower levels of impulsivity than non-meditators. Furthermore, we hypothesized that changes in impulsivity after mindfulness training would be accompanied by changes in the neural correlates of impulsivity, and that long-term meditators would have differences in these neural correlates compared to non-meditators.

To test these hypotheses, this study first examined the effect of an 8-week mindfulness intervention on impulsivity and its neurobiological correlates in healthy adults, and compared changes over time to both an active control group and a waitlist control group. We then examined whether impulsivity and its neurobiological correlates differed in long-term meditators (LTMs; *n* = 28) compared to meditation-naïve participants (MNPs; *n* = 105). Specifically, we examined impulsivity with performance on a task of behavioral inhibition (go/no-go task) and with self-ratings of attentional, motor, and non-planning impulsivity using the Barratt Impulsiveness Scale (BIS-11). The neurobiological correlates of interest were derived from a previous study of the MNPs in this sample^17^, in which less gray matter volume in prefrontal regions, heightened resting-state functional connectivity in basal ganglia-thalamo-cortical circuitry, and lower spontaneous eye-blink rate (indicative of lower central dopamine levels) were predictive of higher levels of impulsivity at baseline.

## Methods

### Overview

At Time 1, 105 meditation naive participants (MNPs) and 31 long-term meditators (LTMs) were compared on levels of impulsivity (BIS-11 scores; go/no-go task performance) and on neurobiology (gray matter volume; resting-state functional connectivity; spontaneous eye-blink rate).

**Figure 1:**
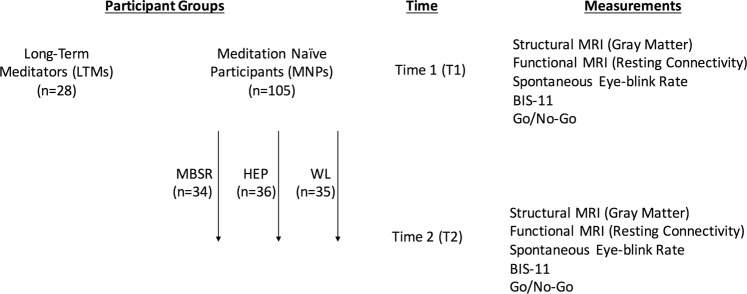
Participant Flow.

Subsequently, MNPs were then randomized into one of three groups (as described previously^41^): an 8-week mindfulness-based stress reduction (MBSR; *n* = 34) intervention group, an 8-week Health Enhancement Program (HEP; *n* = 36) active control group, or a passive waiting list control group (WL; *n* = 35). After the intervention period - Time 2 - all of the impulsivity and neurobiological metrics were measured again for MNPs in the three groups. Group by time analyses were then conducted to determine whether subjects in the MBSR group experienced impulsivity reductions or neurobiological changes - between Time 1 and Time 2 - of a greater magnitude than subjects in the HEP and WL groups did. See Fig. 1 for a visual overview of the participant flow.

### Participants: recruitment and demographics of MNPs and LTMs

Participant recruitment, exclusion criteria and demographics have been previously described^17^. Participants provided written informed consent for study procedures that were approved by the UW-Madison Health Sciences Internal Review Board, and all methods and experiments were performed in accordance with the relevant guidelines and regulations. Meditation-naive participants (MNPs) were recruited for a study on health and well-being through advertisements in Madison, WI, area newspapers, e-mails, and through postings and discussions with meditation teachers and groups. Long-term meditators (LTMs) were recruited in the United States at meditation centers and through related mailing lists, in addition to flyers and advertisements in newspapers. Criteria for exclusion for all participants included use of psychotropic or steroid drugs, night-shift work, diabetes, peripheral vascular disease or other diseases affecting circulation, needle phobia, pregnancy, current smoking habit, alcohol or drug dependency. Participants were also excluded if they had used medication for anxiety, depression, or other psychological issues, or had a psychiatric diagnosis in the past year, and were excluded if they had any history of bipolar or schizophrenic disorders, brain damage or seizures. Additional exclusion criteria for MNPs included significant previous experience with meditation or other mind–body techniques (i.e. if they had ever taken an MBSR course, had meditated or practiced other mind-body techniques at least 3x per week for the past 2 months, or do daily yoga or tai-chi practice), remarkable exercise habits (engagement in moderate sport or recreational activities >5 h per week; engagement in vigorous sport or recreational activities >4 h per week), inability to walk, and inability to attend weekly class and full-day group sessions. Meditation recruitment criteria for LTMs included at least 3 years of daily meditation practice (at least 30 min per day), and at least three intensive retreats lasting 5 days or more.

We also employed a biased coin procedure, based on Frane (1998)^42^, to match the MNP and LTM groups based on age and gender. Specifically, we matched potential MNPs to LTMs on age and gender, only enrolling people into the MNP group when doing so did not result in significant group differences with the LTM group.

In total, 158 healthy human subjects were recruited, which comprised 127 meditation-naive participants and 31 long-term meditators. Structural and functional magnetic resonance imaging (MRI) scans were obtained for 106 MNPs and for 28 LTMs, and so all analyses for the present study were conducted within this set of subjects. One MNP’s scan was excluded due to poor image registration. Thus, the final sample for this study included data from a total of 105 MNPs [age, 48.6 ± 10.9 years (mean ± SD); 65 women, 40 men] and 28 LTMs [age, 49.8 ± 10.1 years (mean ± SD); 15 women, 13 men] Subject demographics and assessment of group differences in demographic variables are detailed in Tables 1, 2. The MNP and LTM groups did not significantly differ in age, gender, household income, or race.

**Table 1:** MNP subgroup demographics.

	MBSR (*n* = 34)		HEP (*n* = 36)		WL (*n* = 35)		
	*Mean*	*SD*	*Mean*	*SD*	*Mean*	*SD*	*p* ^*a*^
*Age*	50.3	9.5	47.9	12.5	47.8	10.5	0.57
*Raven IQ*	51.8 (n = 20)	4.0	53.4 (n = 24)	1.9	52.8 (n = 25)	3.5	0.28
*Household Income (scaled)*	11.3	4.8	12.7	4.0	12.43	3.7	0.34
	%	n	%	n	%	n	
***Gender***							
*Female*	64.7	22	55.6	20	65.7	23	0.63
*Male*	35.3	12	44.4	16	34.3	12	0.63
***Race***							
*Caucasian*	91.2	31	94.4	34	91.4	32	0.85
*Asian*	2.9	1	2.8	1	0	0	0.61
*American Indian or Alaska Native*	2.9	1	0	0	0	0	0.36
*Mixed*	2.9	1	2.8	1	2.9	1	0.99
*Did Not Report*	0	0	0	0	5.7	2	0.13

**Table 2:** LTM and MNP demographics.

	All MNPs (*n* = 105)		LTMs (*n* = 28)		
	*Mean*	*SD*	*Mean*	*SD*	*p* ^*a*^
*Age*	48.6	10.9	49.8	10.1	0.62
*Raven IQ*	52.7 (n = 69)	3.2	52.1 (n = 27)	4.6	0.50
*Household Income (scaled)*	12.2	4.2	11.5	5.3	0.50
	%	n	%	n	
***Gender***					
*Female*	61.9	65	53.6	15	0.43
*Male*	38.1	40	46.4	13	0.43
***Race***					
*Caucasian*	92.4	97	89.2	25	0.60
*Asian*	1.9	2	7.1	2	0.15
*American Indian or Alaska Native*	1.0	1	0	0	0.61
*Mixed*	2.9	3	3.6	1	0.85
*Did Not Report*	1.9	2	0	0	0.47

### Participants: LTMs

LTMs, as previously described^41^, had an average of 9,154 lifetime hours of meditation practice, ranging from 1,439 to 29,046 total hours. Lifetime hours of practice were calculated based on subjects’ reports of their average hours of formal (sitting and walking) meditation practice per week and the total years of practice, including time spent in meditation retreats. All LTM participants were proficient in meditation practices as taught within the framework of either Theravada or Tibetan Buddhism. These practices included two attention-based meditations, which we referred to as open monitoring (OM) and focused attention (FA) meditations, as well as one compassion/loving-kindness meditation referred to as Metta meditation. Briefly, FA meditation involves directing and sustaining attention on a selected object (e.g., breathing), detecting mind wandering and distractors (e.g., thoughts) as well as disengagement of attention from distractors, and shifting of the focus of attention back to the selected object. By contrast, OM meditation has no explicit focus of attention, but rather requires nonreactive meta cognitive monitoring of anything that is experienced, thus replacing the “effortful” selection of an object as primary focus with an “effortless” sustained awareness of the rich features of each experience^43^. The practice of compassion/loving-kindness meditation is a form of concentration practice where the practitioner focuses his/her mind on the suffering of oneself or others and then on the wish that the individual(s) in question may be happy and free from suffering.

### Participants: MNP intervention groups

We took all the MNPs enrolled and again used a biased coin procedure to determine MNP group assignment, matching age and gender in three intervention groups: an 8-week mindfulness-based stress reduction (MBSR; *n* = 34) class, an 8-week Health Enhancement Program (HEP; *n* = 36) as an active control group, or a no-intervention waiting list control group (WL; *n* = 35). MBSR training consists of continuous focused attention on the breath, bodily sensations, and mental content while in seated postures, walking, and yoga^44^. This program can be conceptualized as incorporating OM-related meditations with FA- related meditations. In order to isolate the effects of mindfulness, we designed an active comparison intervention to control for the aspects of MBSR that are known to promote positive outcomes but are not specific to mindfulness, such as a supportive group atmosphere, expert instruction, and engaging in activities that are believed to provide benefit. Our active comparison condition—HEP— matched MBSR in structure, instructor expertise, and content (see (^45^ for more detailed information). HEP consisted of four components: (1) physical activity (e.g., walking); (2) balance, agility, and core strength; (3) nutritional education; and (4) music therapy. Each of these components was chosen to match the collateral benefits that MBSR may produce that are not unique to mindfulness. For example, physical activity with a focus on walking was selected to control for the physical benefits of walking meditation. Each component was delivered by an expert in the respective practice, over eight weekly 2.5-h sessions and one full-day session. Like those participating in MBSR training, HEP participants were assigned 45 to 60 min of daily at-home practice.

### Impulsivity measures

#### Barratt impulsiveness Scale (BIS-11)

As previously described^17^, the BIS-11^46^ is a self-report questionnaire containing 30 questions, each of which requires the subject to choose between ‘Rarely/Never’, ‘Occasionally’, ‘Often’ and ‘Almost Always’. Items are scored from 1 to 4. Scoring yields a total score and three subscale scores derived by factor analysis: attentional impulsivity (e.g. “I am restless at the theatre or lectures”), motor impulsivity (e.g. “I do things without thinking”), and non-planning impulsivity (e.g. “I am more interested in the present than the future”)^47^. Higher scores indicate higher levels of impulsivity. Subjects whose total scores reach above 71 are considered to be “highly impulsive”^48^. The BIS-11 has good internal consistency (Cronbach’s α = 0.83) and test–retest reliability (Spearman’s rho = 0.83)^48^. However, more recent evaluations have indicated some potential issues with the BIS-11, including a lack of evidence for its three-factor structure, redundancy of items and low correlations between items^49,50^. As such, we supplemented our assessment of impulsivity with a task-based measure, the go/no-go task.

BIS-11 data were obtained for 105 of the 105 MNPs and for 28 of the 28 LTMs. At Timepoint 1, six HEP subjects and one LTM subject had BIS-11 total scores above 71, and at Timepoint 2, seven HEP subjects and one LTM subject had BIS-11 total scores above 71, indicating highly impulsive subjects, but whose scores were not outliers in the distribution of scores in this sample.

#### Go/No-Go task

As previously described^17^, subjects completed an auditory go/no-go task based on the paradigm described in Shalgi *et al*.^51^. Subjects were instructed to push the spacebar on a keyboard upon the presentation of an auditory syllable stimulus, except when the same syllable was repeated (no-go repeat trials) or when the syllable was “ke/“ or “pa/“ (no-go syllable trials). Subjects completed four blocks of 252 trials, of which 196 trials were go trials, 16 were no-go repeat trials, and 40 were no-go syllable trials. Accuracy was calculated as the percentage of correct button-pushes for go-trials and the percentage of correct withholds for no-go trials. Post-error slowdown was calculated as the difference between average reaction time on go-trials following incorrect no-go trials and average reaction time on go-trials following correct no-go trials.

As previously described^17^, previous studies have shown that inhibitory capacity on no-go trials is sensitive to task demands, and that different task demands recruit distinct sets of brain regions and cognitive functions. For instance, Shalgi and colleagues find that subjects perform better on no-go syllable trials than no-go repeat trials^51^. This is consistent with a meta-analysis that classifies go/no-go tasks in which the no-go stimuli are constant (as in the no-go syllable trials) as “simple”, and classifies go/no-go tasks in which the no-go stimuli change depending on context (as in the no-go repeat trials) as “complex”^52^; this study also found that complex no-go trials recruit prefrontal regions to a greater extent than simple no-go trials, likely due to the increased attentional and working memory loads required for these trials. Given these performance and neurobiological differences, we analyzed accuracy on each type of no-go trial separately.

Go/no-go data were obtained for 105 of the 105 MNPs and 26 of the 28 LTMs [age, 48.8 ± 9.9 years (mean ± SD); 13 women, 13 men]. One LTM did not complete the go/no-go due to a scheduling issue, and one LTM opted not to continue to the test portion of the task after the practice trials.

### Neurobiological measures

#### Image acquisition

As previously described^17^, images were acquired on a GE X750 3.0 Tesla MRI scanner device with an eight-channel head coil. Anatomical scans consisted of a high-resolution 3D T1-weighted inversion recovery fast gradient echo image (inversion time = 450 ms, 256 × 256 in-plane resolution, 256 mm FOV, 124 × 1.0 mm axial slices). Resting-state functional images were acquired in a single scan run using a gradient echo EPI sequence (64 × 64 in-plane resolution, 240 mm FOV, TR/TE/Flip = 2000ms/25 ms/60°, 40 × 4 mm interleaved sagittal slices, and 210 3D volumes).

### Structural MRI

#### Preprocessing

As previously described^17^, preprocessing of structural MRI data were conducted in Statistical Parametric Mapping software (SPM12; http://www.fil.ion.ucl.ac.uk/spm). For preprocessing, T1 images were first manually realigned; manual realignment involved adjusting the pitch, roll and yaw of each T1 such that the anterior commissure and posterior commissure were in the same axial plane in the sagittal view, and that the midlines in the coronal and axial views followed vertical lines. T1 images were then segmented into gray matter, white matter, and cerebrospinal fluid; a study-specific average template was then created using the DARTEL (Diffeomorphic Anatomical Registration using Exponentiated Lie algebra) algorithm^53^, through which T1 images were aligned and normalized to Montreal Neurological Institute (MNI) space; images were then modulated after normalization to preserve volume; and smoothed with an 8 mm full-width at half-maximum (FWHM) Gaussian kernel^54^.

#### Analysis

Voxel-based morphometry (VBM) in SPM12 was used to assess differences in gray matter volume. Significance for whole-brain voxel-wise volumetric analyses was evaluated using family wise error (FWE) cluster correction. The cluster extent threshold corresponded to the statistical probability (α = 0.05, or 5% chance) of identifying a random noise cluster at a predefined voxel-wise (i.e., whole-brain) threshold of *p* < 0.001 (uncorrected). We used 3dClustSim (updated December 2015) to determine that a cluster-corrected size of ≥236 voxels was significant at *p*_FWE_ < 0.05.

### Resting-state fMRI

#### Preprocessing

As previously described^17^, resting-state data were processed using a combination of FEAT (FMRI Expert Analysis Tool) Version 6.00, part of FSL (FMRIB’s Software Library, www.fmrib.ox.ac.uk/fsl) and AFNI^55^. We removed the first four volumes from each subject’s data, then used FSL’s FEAT tool for motion correction with MCFLIRT^56^, and non-brain removal using BET^57^. Transformation matrices for registration were computed and applied using FSL to register the subject’s time series data to their anatomical template, and a 12DOF affine transformation was used to register the subject’s anatomical to Montreal Neurological Institute (MNI) space using FLIRT^56,58^. Registration from high resolution structural to standard space was then further refined using FNIRT nonlinear registration^59,60^. Images were segmented into white matter, grey matter and cerebrospinal fluid with FAST for use as masks that were eroded using a 3 × 3 × 3 voxel kernel. These and 6 motion regressors of no interest were included in a nuisance regression using AFNI’s 3dDeconvolve. Images were smoothed using a 5 mm FWHM Gaussian kernel.

#### Analysis

Six functionally distinct striatum seeds, each with a radius of 3.5 mm, were created in each hemisphere (for a total of 12 striatal seeds) based on coordinates reported by Di Martino and colleagues (2008)^61^. The origin coordinates (in MNI space) of these seeds were in the inferior ventral striatum (±9, 9, −8), superior ventral striatum (±10, 15, 0), dorsal caudate (±13, 15, 9), dorsal caudal putamen (±28, 1, 3), dorsal rostral putamen (±25, 8, 6), and ventral rostral putamen (±20, 12, −3). Furthermore, a substantia nigra seed in each hemisphere (±12, −12, −12) and midline ventral tegmental area seed (0, −15, −12), each with a radius of 2 mm, were created based on coordinates reported by Tomasi and Volkow (2012)^62^. The striatal seeds were chosen because of their demonstrated functional connectivity to distinct prefrontal-basal ganglia circuits that process unique types of information relevant for decision-making^61^, and the prior demonstration that individual variation in the strength of these circuits is related to individual variation in impulsivity^17^. The midbrain seeds were chosen because they are sites of dopaminergic projection neurons that innervate the basal ganglia and prefrontal cortex, and the strength of these circuits has also been implicated in contributing to individual variability in impulsivity^17^. Collectively, seeding these 6 striatal and 2 midbrain regions allows for holistic examination of distinct cortico-subcortical circuits thought to play a role in individual variability in impulsivity. A primary visual cortex seed was used as a control region^63^.

Average time-series were extracted for each seed from each participant’s preprocessed data. These timeseries were regressed back onto each participant’s data using 3dDeconvolve. To further address motion, high motion time points (a frame-wise displacement (FD) measure larger than 0.2 mm) were removed. See Table S1 for the number of FDs > 0.2 mm in each participant group. Participants with more than 25% (52 TRs) of the data censored were omitted from resting-state functional connectivity (RSFC) analysis, leading to a total of 33 excluded participants. 27 of the 105 MNPs and six of the 28 LTMs were excluded, and so data from 78 MNPs [age, 48.9 ± 11.0 (mean ± SD); 50 women, 28 men] and 22 LTMs [age, 48.8 ± 11.5 (mean ± SD); 11 women, 11 men] were available for RSFC analyses. Subjects excluded from RSFC analyses did not significantly differ from subjects included on any demographic measures (Table S2).

The resultant maps were Fisher-Z transformed to stabilize correlation variance. Voxel-wise, group-level analyses of seed-to-wholebrain connectivity maps were conducted for each seed using FSL’s Randomize^64^ thresholded at *p* < 0.05 controlling for family-wise error using threshold-free cluster enhancement with 5000 permutations.

### Spontaneous eye-blink recording and preprocessing

Our protocol for sEBR recording and preprocessing is as previously described^41^. Since spontaneous eye-blink rates are affected by the time of day^65^, data were collected around 7 pm for all participants to ensure that differences in the time of data collection could not contribute to any observed difference in eye-blink activity. Baseline sEBR was extracted from high- density, 256-channel EEG data that were collected during a 10-min baseline EEG recording. Participants were seated in front of a computer screen. During the first 2 min and last 2 min of the baseline recording, participants were instructed to keep their eyes closed. During the 6 min in between these periods, participants were instructed to keep their eyes open while looking at a cross in the middle of a fixation screen. No explicit instruction was given about blinking behavior to insure its spontaneity. Eye-blink data were extracted from the 6-min baseline recording with eyes open. Artifacts and bad channels (i.e., channels with high impedance/poor contact with the scalp) were removed from the raw EEG data using EEGLAB, and a low-pass filter of 100 Hz was applied before data analysis. After performing an independent component analysis (ICA) in MATLAB, maximally independent components were selected based on the presence of eye-blink activity, its temporal activity, and its frontal distribution. Based on the time points of the individual eye-blinks, sEBR per minute was computed. The vertical eye-blink power spectrum is concentrated in the range 0.5 to 3 Hz. There, the power of blinks is in the order of 10 times larger in amplitude than the average cortical signals, and lasts for approximately 300 ms^66^. These particular characteristics enable reliable statistical separation of eye-blink-related signals from brain-related or EMG-related signal from the EEG signals. The amplitude threshold for peak detection was verified manually for every participant and manually adapted if needed to assure correct quantification of eye-blink rates.

Spontaneous eye blink rate (sEBR) data were obtained for 91 of the 105 MNPs [age, 48.6 ± 10.9 years (mean ± SD); 54 women, 37 men] and for 24 of the 28 LTMs [age, 49.7 ± 9.9 years (mean ± SD); 13 women, 11 men].

## Results

### Intervention

In order to assess whether MBSR training had an effect on impulsivity or neurobiological measures, a two-way repeated measures ANOVA was performed for each measure, with time as within-subject factor (pre-intervention Time 1 vs. post-intervention Time 2) and group as between-subjects factor (WL, HEP, MBSR). All analyses control for age and gender (gray matter volume analysis additionally controls for intracranial volume). The inclusion or exclusion of outcome variable outliers (defined as values more than 3 standard deviations from the within-group mean) did not affect the results. As such, results are reported with outliers included. Results of the analyses with outliers excluded can be found in Table S3.

#### BIS-11 self-report

**Figure 2:**
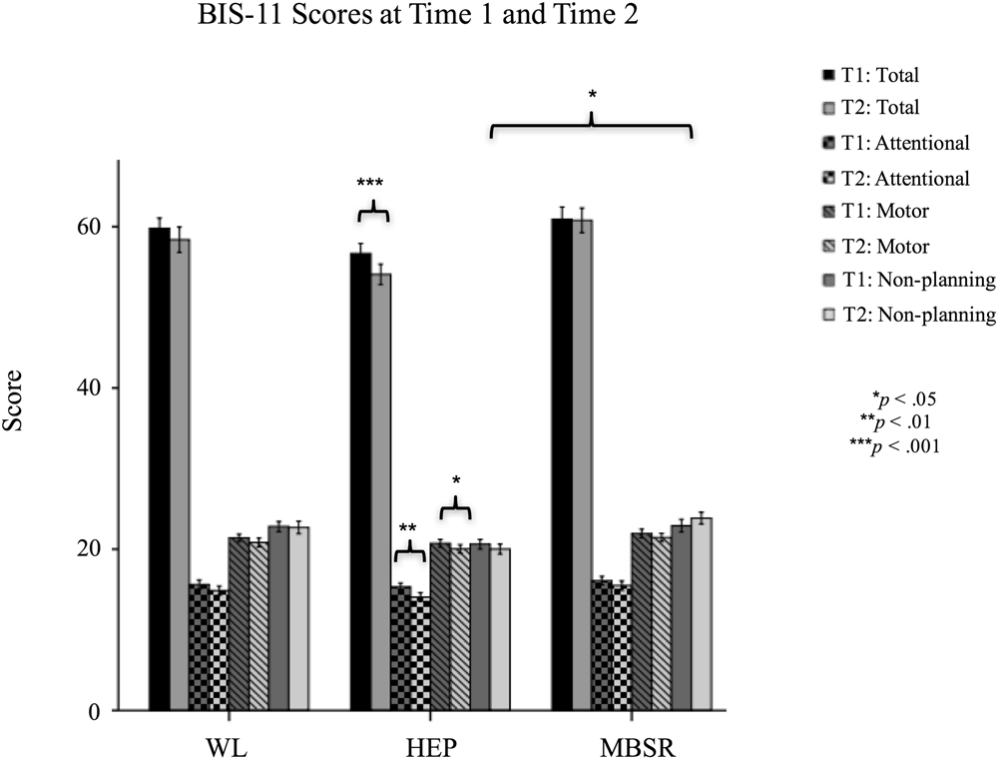
BIS-11 Scores at Time 1 and Time 2.

The overall group-by-time interaction was found to be non-significant for attentional impulsivity score, F(2,99) = 0.577, *p* = 0.563, motor impulsivity score, F(2,99) = 0.049, *p* = 0.952, and total impulsivity score, F(2,99) = 1.826, p = 0.166, but was trending for non-planning impulsivity score, F(2,99) = 3.078, *p* = 0.050. Post-hoc analysis showed that this interaction was driven by non-significant decreases in non-planning impulsivity score from Time 1 to Time 2 in WL and HEP subjects but a non-significant increase in score from Time 1 to Time 2 in MBSR subjects. Within the MBSR group, none of the BIS-11 score-types significantly decreased from Time 1 to Time 2, whereas within the HEP group, total impulsivity score, F(1,35) = 14.942, *p* < 0.001, attentional impulsivity score, F(1,35) = 11.741, *p* = 0.002, and motor impulsivity score, F(1,35) = 6.631, *p* = 0.014, significantly decreased from Time 1 to Time 2. None of the BIS-11 score-types significantly decreased in WL subjects from Time 1 to Time 2. See Fig. 2, Supplemental Table S4.

#### Go/No-Go task

**Figure 3:**
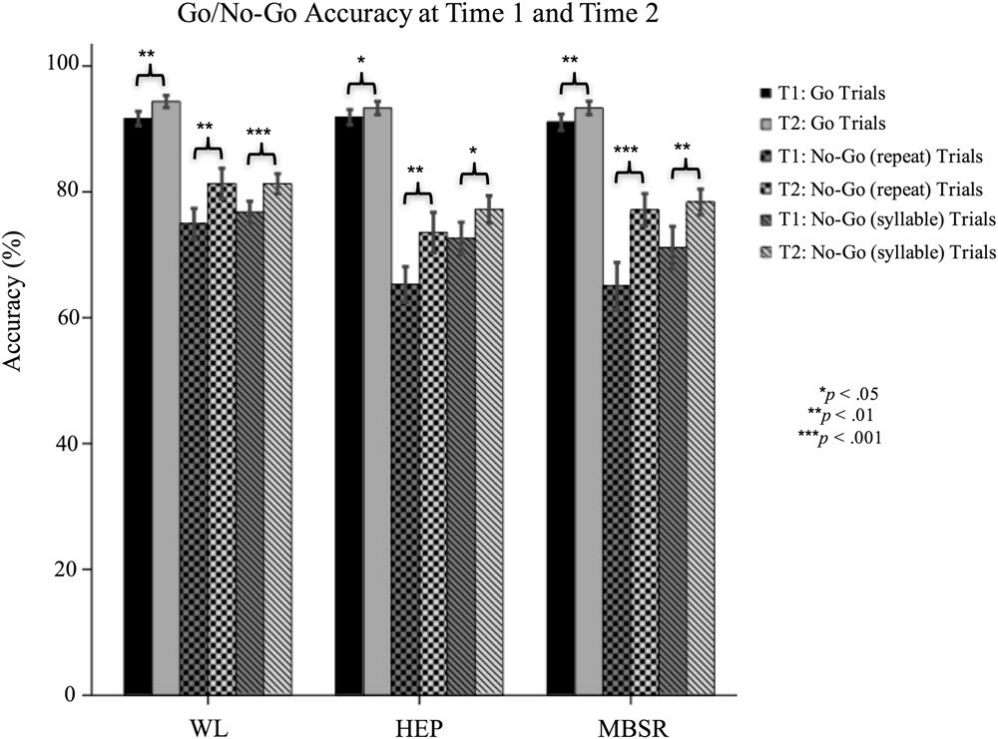
Go/No-Go Accuracy at Time 1 and Time 2.

**Figure 4:**
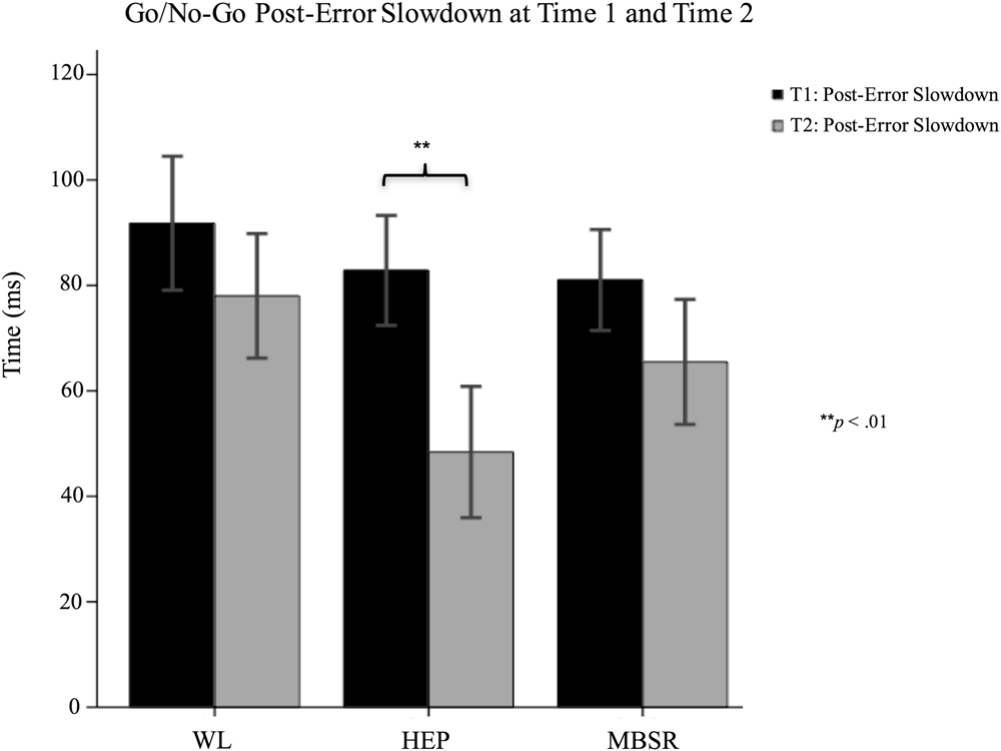
Go/No-Go Post-Error Slowdown at Time 1 and Time 2.

The overall group-by-time interaction was found to be non-significant for go trial accuracy, F(2,99) = 0.547, *p* = 0.580, no-go repeat trial accuracy, F(2,99) = 1.113, *p* = 0.333, no-go syllable trial accuracy, F(2,99) = 0.736, *p* = 0.482, and post-error slowdown, F(2,99) = 1.082, *p* = 0.343. Post-hoc analyses showed that accuracy on all three trial-types significantly increased from Time 1 to Time 2 in all three groups. Post-error slowdown decreased from Time 1 to Time 2 in all three groups, but this decrease was only significant for the HEP group, F(1,35) = 8.005, *p* = 0.008. See Figs 3 and 4.

#### Gray matter volume

Whole-brain VBM analyses revealed no significant differences between groups in the change in gray matter volume from Time 1 to Time 2.

To corroborate these null findings, supplementary surface-based cortical thickness analyses were conducted in FreeSurfer. These analyses also yielded null findings, with the exception of a MBSR vs. WL difference in postcentral gyrus, but this was driven by a change within the WL group. See Supplemental Analysis 1.

#### Resting-state functional connectivity

No significant differences were found between groups in the change in RSFC from Time 1 to Time 2 between any of the seed regions and the rest of the brain.

#### Sponthaneous eye-blink rate

**Figure 5:**
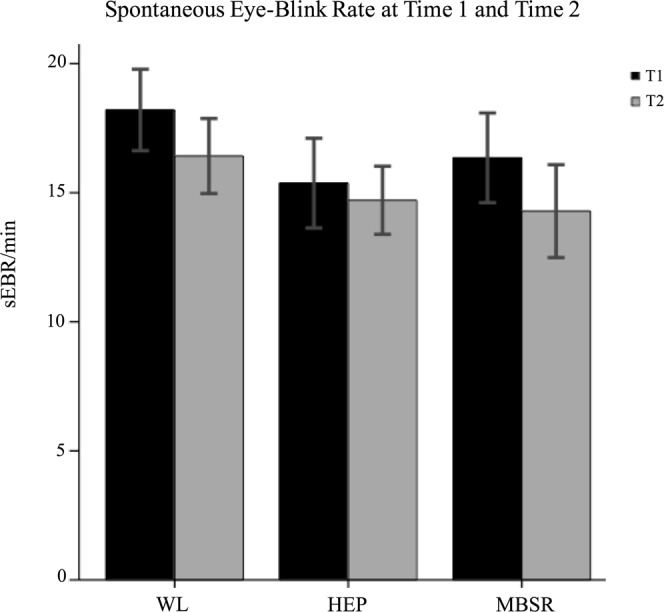
Spontaneous eye-blink rate at Time 1 and Time 2.

Findings from a group-by-time interaction in relation to sEBR have previously been reported in a superset of this sample by Kruis and colleagues^41^, in which no significant interaction was found. As this sample differs slightly from the one previously reported on, we repeated the analysis here in order to ensure that the relationship holds in the present sample. Indeed, we found that the interaction was non-significant, F(2,86) = 0.137, p = 0.872, and post-hoc analyses found that sEBR did not change significantly from Time 1 to Time 2 in any of the three groups. See Fig. 5.

#### Post-hoc analysis: baseline factors predictive of intervention-related change

We examined whether any baseline factors (i.e. age, gender, and baseline BIS-11 total score) predicted greater change in BIS-11 total score after the intervention. We found that across all MNPs there was a gender effect, such that males experienced significantly greater reductions in BIS-11 total score than females (B = 2.1 points) between Time 1 and Time 2, F(1,100) = 4.7, p = 0.032. This was driven by the HEP group, the only group where this effect was statistically significant; on average, males experienced a reduction in BIS-11 total scores that was B = 3.1 points more than females did, F(1,32) = 6.7, p = 0.014. Age and baseline BIS-11 total score were not predictive of intervention-related change.

#### Post-hoc analysis: home practice hours

MBSR and HEP participants logged their daily home practice hours. MBSR participants completed a total average of 2320 minutes of home practice (range 234 to 5111). HEP participants completed a total average of 3867 minutes of home practice (range 1581 to 15291). The difference in mean home practice time between groups was statistically significant, F(1,68) = 11.1, p = 0.001. However, neither across groups (p = 0.915) nor within either group (p = 0.118 for HEP, p = 0.571 for MBSR) was home practice hours associated with change in BIS-11 total scores between Time 1 and Time 2, controlling for age, gender, and baseline BIS-11 total score. Without these covariates, the relationship within the HEP group becomes significant, F(1,35) = 7.387, p = 0.010. However, when this analysis is split by gender in the HEP group, it reveals that the relationship is only significant for women: practice hours were *negatively* associated with change in BIS-11 score (i.e. more practice hours were associated with less of a decrease in impulsivity), F(1,18) = 6.458, p = 0.020 (F(1,14) = 0.161, p = 0.694 for males). Within the HEP group, females completed more practice hours than males on average, but the difference was not significant, F(1,34) = 0.819, p = 0.372.

#### Post-hoc analysis: the relation of changes in impulsivity to changes in mindfulness

To examine whether the HEP group’s decrease in BIS-11 impulsivity after the intervention was related to a change in mindfulness after the intervention, we analyzed the correlation between BIS-11 change and mindfulness change (as measured by the Five Factor Mindfulness Questionnaire) between Time 1 and Time 2. This correlation (r = 0.045) was not significant (*p* = 0.794), nor was it significant when controlling for age and gender (*p* = 0.881).

### LTMs vs. MNPs

All LTM versus MNP group comparisons use t-test analysis of the Time 1 data, and control for age and gender (gray matter volume analysis additionally controls for intracranial volume). The inclusion or exclusion of outcome variable outliers (defined as values more than 3 standard deviations from the within-group mean) did not affect the results, except for one analysis where this is noted. As such, results are reported with outliers included; results of the analyses with outliers excluded can be found in Supplementary Table S3.

#### BIS-11 self-report

**Figure 6:**
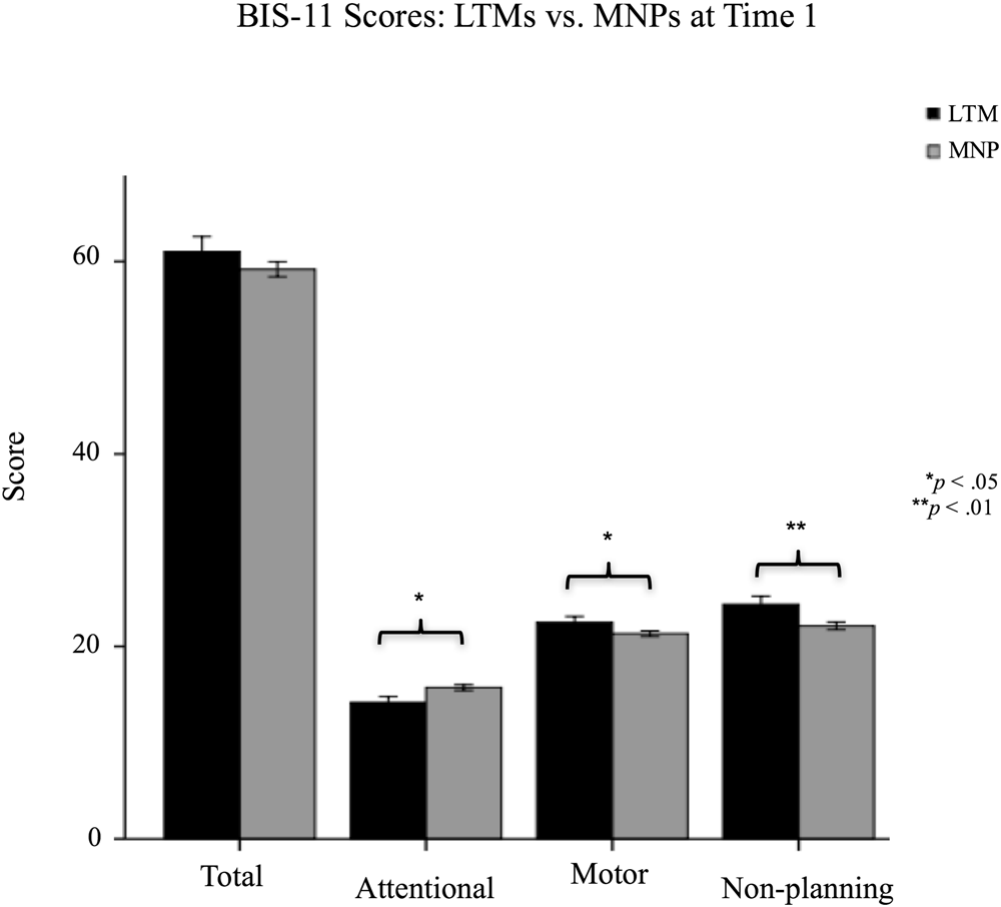
BIS-11 Scores: LTMs vs. MNPs.

Compared to MNPs, LTMs scored lower (less impulsive) on the attentional impulsivity subscale, F(1,129) = 5.005, *p* = 0.027, B = −1.51, but higher (more impulsive) on the motor impulsivity subscale, F(1,129) = 3.938, *p* = 0.049, B = 1.31, and non-planning impulsivity subscale, F(1,129) = 7.115, *p* = 0.009, B = 2.36. Groups did not differ significantly on overall score, F(1,129) = 1.527, *p* = 0.219, B = 2.15). See Fig. 6.

#### Go/No-Go task

**Figure 7:**
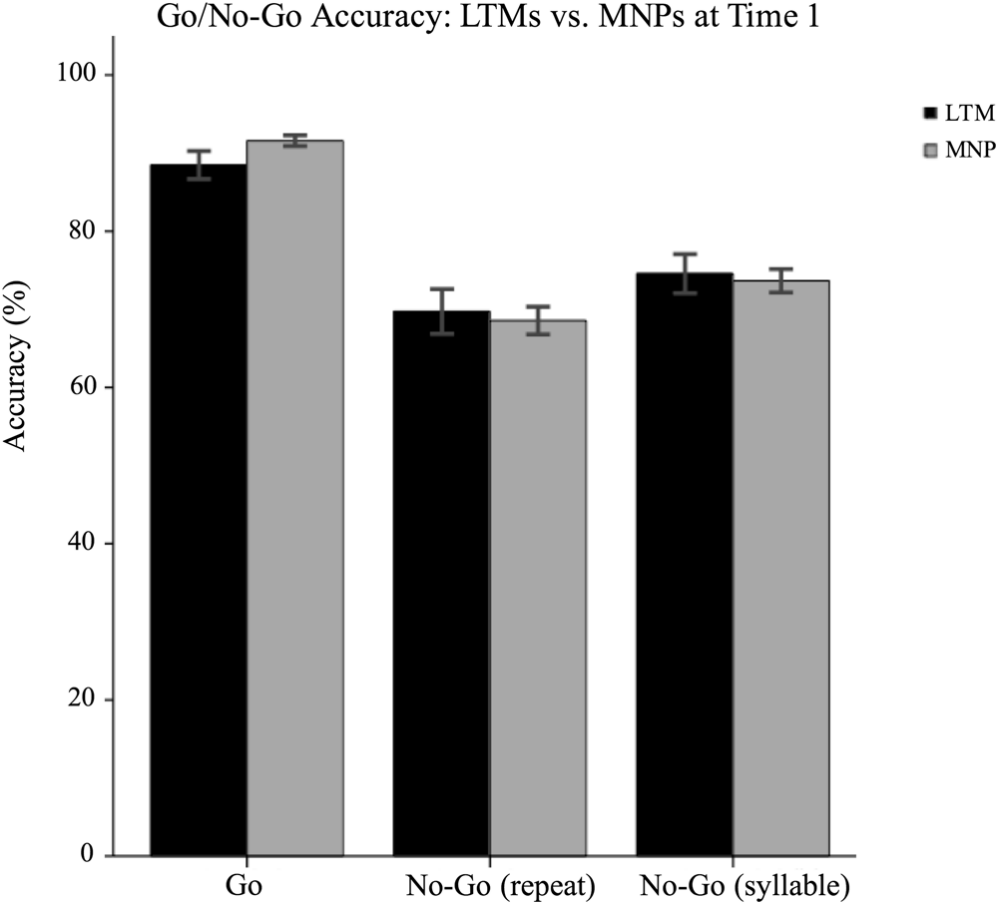
Go/No-Go Accuracy: LTMs vs. MNPs.

**Figure 8:**
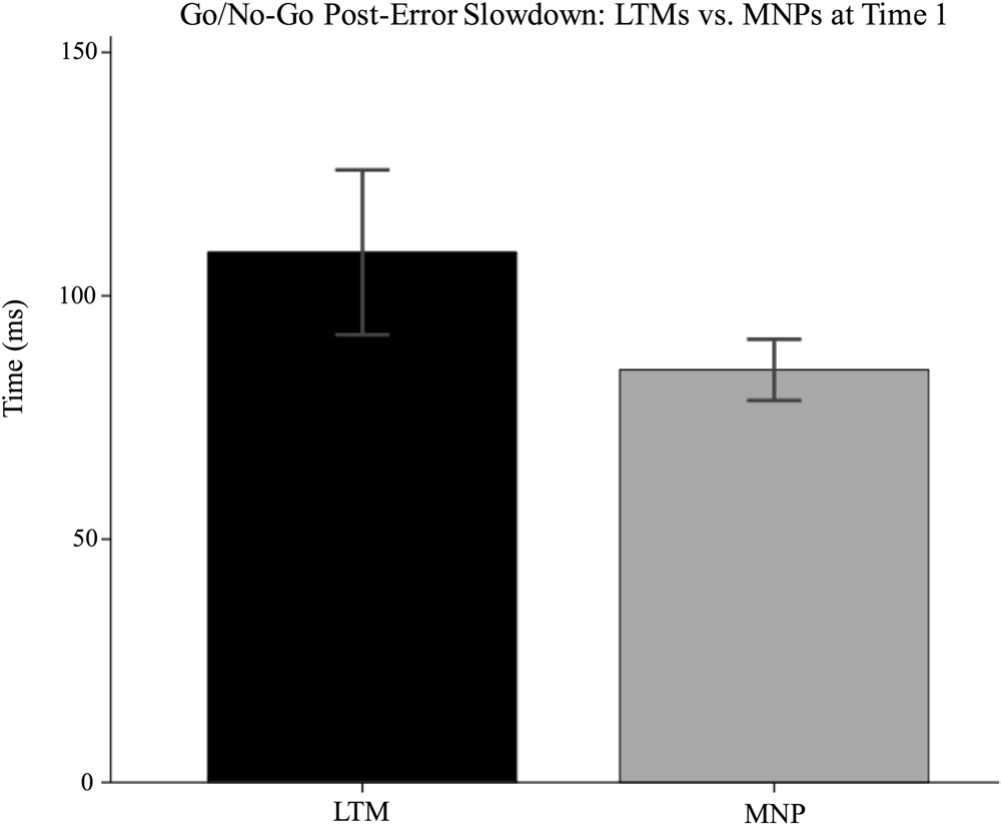
Go/No-Go Post-Error Slowdown: LTMs vs. MNPs.

LTMs did not differ significantly from MNPs on no-go (repeat) trial accuracy, F(1,127) = 0.165, *p* = 0.685, B = 1.57, no-go (syllable) trial accuracy, F(1,127) = 0.252, *p* = 0.617, B = 1.61, or post-error slowdown, F(1,127) = 2.299, *p* = 0.132, B = 23.1. See Figs 7 and 8. On go trial accuracy, a group difference of trending significance, F(1,127) = 3.595, *p* = 0.060, B = −3.1, became significant after removal of an outlier, F(1,126) = 4.345, p = 0.039, B = −3.32, in which MNPs were more accurate than LTMs.

#### Gray matter volume

**Figure 9:**
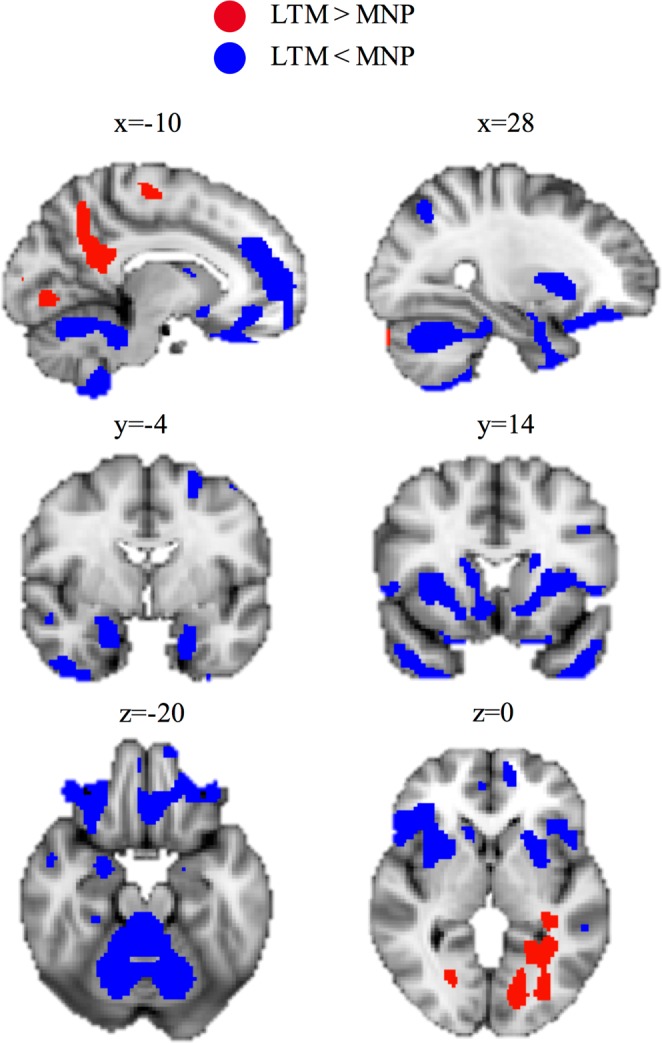
Gray Matter Volume: LTMs vs. MNPs.

Whole-brain analyses showed that LTMs had less gray matter volume than MNPs in significant clusters encompassing parts of bilateral medial orbitofrontal cortex, inferior frontal gyrus, paracingulate gyrus, parahipocampal gryus, temporal pole, striatum, amygdala and cerebellum, as well as left dorsolateral prefrontal cortex and precentral gyrus. On the other hand, LTMs were found to have more gray matter volume than MNPs in significant clusters encompassing parts of the precuneus, posterior cingulate cortex, precentral gyrus, fusiform gyrus, angular gyrus and middle temporal gyrus. See Tables 3, 4 and Fig. 9 for full results.

**Table 3:** Regions with less volume in LTMs compared to MNPs.

Region	Other Regions in Cluster	Cluster Size	MNI Peak Coordinates
R Frontal Operculum Cortex	R Inferior Frontal Gyrus, R Medial Orbitofrontal Cortex, R Lateral Orbitofrontal Cortex, R Putamen	4972	(48, 24, 2)
L Cerebellum	R Cerebellum	17477	(−34, −54, −33)
L Middle Frontal Gyrus	L Superior Frontal Gyrus	997	(−28, 2, 57)
L Medial Orbitofrontal Cortex	L Paracingulate Gyrus, R Paracingulate Gyrus, R Subcallosal Cortex, R Frontal Pole, R Inferior Frontal Gyrus, R Frontal Operculum Cortex, R Nucleus Accumbens, L Nucleus Accumbens, R Caudate, L Caudate, R Putamen, L Putamen, R Insula, L Insula	7137	(−10, 32, −30)
L Middle Frontal Gyrus	L Precentral Gyrus	534	(−39, 9, 32)
R Amygdala	R Parahippocampal Gyrus, R Temporal Pole	995	(24, −2, −20)
R Inferior Temporal Gyrus		443	(45, −9, −46)
L Middle Frontal Gyrus		440	(−30, 0, 57)
L Temporal Pole	L Parahippocampal Gyrus, L Amygdala	527	(−26, 3, −46)

**Table 4:** Regions with more volume in LTMs compared to MNPs.

Region	Other Regions in Cluster	Cluster Size	MNI Peak Coordinates
L Precuneus	L Posterior Cingulate Gyrus	2366	(−15, −56, 45)
L Precentral Gyrus		339	(−16, −15, 56)
L Middle Temporal Gyrus	L Fusiform Gyrus, L Lingual Gyrus	2418	(−48, −50, −4)
L Lingual Gyrus	L Occipital Pole	906	(−14, −80, −4)
L Cerebellum		342	(−16, −60, −44)
R Lingual Gyrus	R Fusiform Gyrus	392	(21, −69, −4)

#### Resting-state functional connectivity

**Figure 10:**
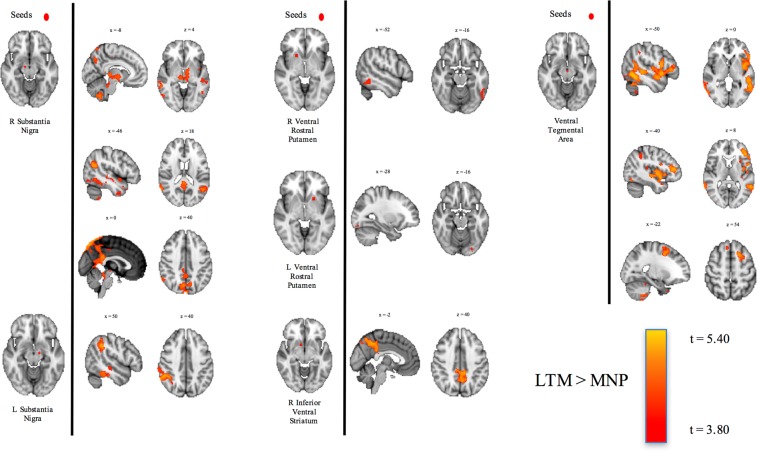
Resting-State Functional Connectivity: LTMs vs. MNPs.

Compared to MNPs, LTMs had increased positively correlated RSFC between the ventral tegmental area and areas within the inferior and superior frontal gyri, between the substantia nigra and globus pallidus, thalamus, and default mode network nodes such as the precuneus and angular gyrii, and between the inferior ventral striatum and default mode network nodes. No significant group differences in RSFC between the control V1 seed and the rest of the brain were observed. See Table 5 and Fig. 10 for full results.

**Table 5:** Greater RSFC in LTMs compared to MNPs.

Focal Seed	RSFC Relationship with:	Other Regions in Cluster	MNI Peak Coordinates	Cluster Size	t-value
R Substantia Nigra	R Precuneus	L Globus Pallidus, R Thalamus, L Thalamus, L Precuneus, L Posterior Cingulate Gyrus, R Posterior Cingulate Gyrus	(6, −76, 52)	3053	4.48
	L Cerebellum	L Midbrain	(−30, −44, −44)	1761	4.26
	L Temporal Pole		(−36, 20, −32)	901	3.86
	R Middle Temporal Gyrus	R Angular Gyrus	(66, −50, 12)	748	4.69
	L Lateral Occipital Cortex	L Angular Gyrus	(−46, −62, 22)	551	4.50
	R Inferior Temporal Gyrus	R Inferior Lateral Occipital Cortex	(52, −56, −16)	333	4.19
L Substantia Nigra	R Angular Gyrus	R Supramarginal Gyrus, R Superior Temporal Gyrus, R Middle Temporal Gyrus, R Inferior Temporal Gyrus	(54, −46, 34)	2220	4.09
R Ventral Rostral Putamen	L Inferior Temporal Gyrus		(−52, −54, −14)	94	3.68
L Ventral Rostral Putamen	L Occipital Fusiform Gyrus		(−28, −86, −16)	13	4.18
R Inferior Ventral Striatum	L Precuneus	R Precuneus, L Posterior Cingulate Gyrus. R Posterior Cingulate Gyrus	(−6, −54, 48)	1275	4.26
Ventral Tegmental Area	L Inferior Temporal Gyrus	L Inferior Frontal Gyrus, L Middle Temporal Gyrus, L Angular Gyrus, L Insula,	(−50, −56, −10)	5050	4.51
	R Inferior Lateral Occipital Cortex		(58, −66, −4)	501	5.05
	L Superior Frontal Gyrus		(−16, 14, 50)	448	5.37
	L Cerebellum		(−18, −50, −52)	241	4.49
	L Supramarginal Gyrus		(−46, −44, 40)	199	3.50
	R Superior Frontal Gyrus		(6, 30, 62)	129	4.45
	L Heschl’s Gyrus		(−36, −28, 10)	89	3.90

#### Spontaneous eye-blink rate

**Figure 11:**
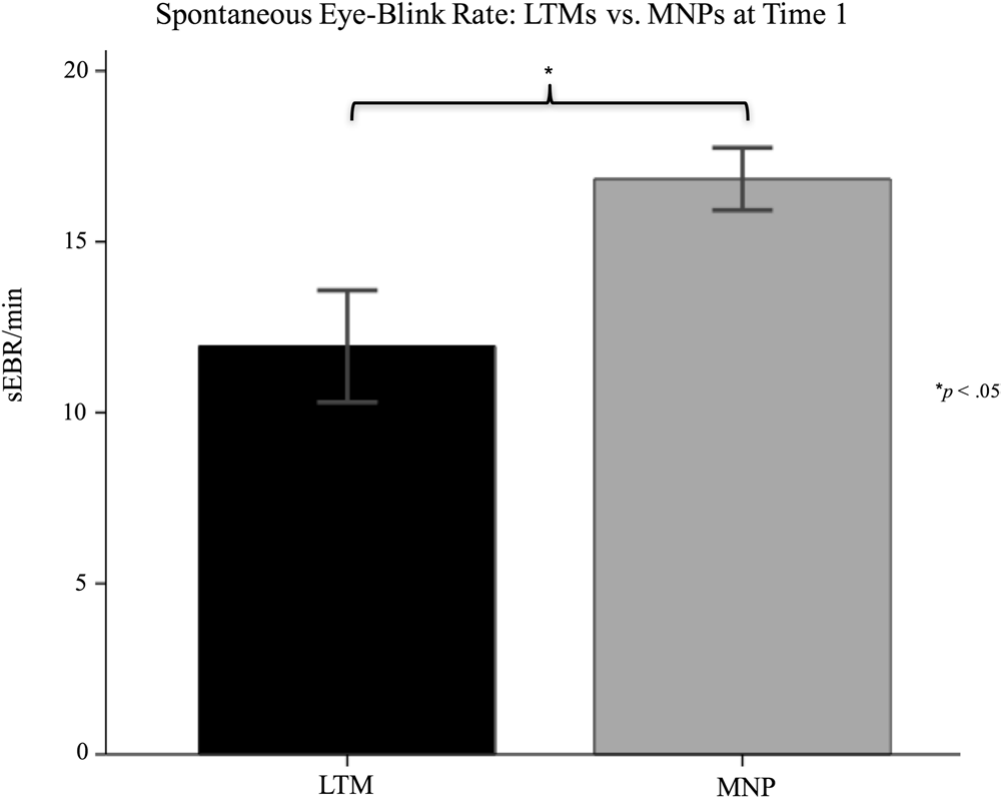
Spontaneous eye-blink rate: LTMs vs. MNPs.

Findings of a group difference in sEBR have previously been reported in a superset of this sample by Kruis and colleagues^41^, with LTMs having been found to have significantly lower sEBR than MNPs. As this sample differs slightly from the one previously reported on, we repeated the analysis here in order to ensure that the relationship holds in the present sample. Indeed, LTMs had significantly lower sEBR than MNPs, F(1,122) = 6.139, *p* = 0.015, B = −4.73. See Fig. 11.

### Practice hours in LTMs

Total lifetime practice hours (TLPH) and total retreat practice hours (TRPH) were log-transformed in order to normalize the data.

#### BIS-11 self-report

logTLPH had non-significant relationships with attentional impulsivity score, F(1,24) = 3.738, *p* = 0.065, B = −1.78, motor impulsivity score, F(1,24) = 0.697, *p* = 0.412, B = 0.823, non-planning impulsivity score, F(1,24) = 0.108, *p* = 0.745, B = 0.427, and total impulsivity score, F(1,24) = 0.259, *p* = 0.829, B = −0.532. logTRPH had non-significant relationships with attentional impulsivity score, F(1,24) = 1.091, *p* = 0.307, B = −1.27, motor impulsivity score, F(1,24) = 0.018, *p* = 0.896, B = 0.17, non-planning impulsivity score, F(1,24) = 0.409, *p* = 0.529, B = 1.03, and total impulsivity score, F(1,24), *p* = 0.983, B = −0.07.

#### Go/No-Go task

logTLPH was not significantly related to go trial accuracy, F(1,22) = 1.366, *p* = 0.255, B = −3.11, no-go repeat trial accuracy, F(1,22) = 2.472, *p* = 0.130, B = 6.39, no-go syllable trial accuracy, F(1,22) = 1.327, *p* = 0.262, B = 4.26, or post-error slowdown, F(1,22) = 1.777, *p* = 0.196, B = 30.90. logTRPH was not significantly related to go trial accuracy, F(1,22) = 2.213 *p* = 0.151, B = −4.86, no-go repeat trial accuracy, F(1,22) = 0.083, *p* = 0.776, B = −1.55, no-go syllable trial accuracy, F(1,22) = 0.285, *p* = 0.599, B = −2.53, or post-error slowdown, F(1,22) = 0.231, *p* = 0.635, B = −14.41.

#### Gray matter volume

Whole-brain analyses did not reveal any significant clusters where logTLPH or logTRPH was related to gray matter volume.

#### Resting-state functional connectivity

Neither logTLPH nor logTRPH was significantly related to RSFC between any of the seed regions and the rest of the brain.

#### Spontaneous eye-blink rate

Neither logTLPH, F(1,22) = 1.138, *p* = 0.297, B = −2.68, nor logTRPH, F(1,24) = 0.220, *p* = 0.643, B = −1.50, was significantly related to sEBR in this sample, consistent with the findings from the superset reported by Kruis and colleagues^41^.

## Discussion

Recent interest has grown in using mindfulness meditation to help treat conditions featuring high levels of impulsivity such as substance abuse and ADHD. However, impulsivity is a multidimensional trait that can arise from deficits in one of several cognitive faculties, such as maintaining attention, inhibiting urges, and/or prospectively planning behavior. While a number of studies find that mindfulness can improve attention^10,11^, it remains unclear whether mindfulness is effective in improving other cognitive faculties whose deficiency can contribute to impulsive behavior. As such, this study first examined the effect of an eight-week mindfulness intervention on dimensions of impulsivity – gauged by performance on a go/no-go task and self-ratings of attentional, motor, and non-planning impulsivity on the Barratt Impulsiveness Scale (BIS-11) – and on gray matter volume and resting-state functional connectivity in the brain’s decision-making circuitry, as well as on spontaneous eye blink-rate (a physiological indicator of dopaminergic functioning). We also examined cross-sectional differences in these metrics between long-term meditators (LTMs; *n* = 28) and meditation-naïve participants (MNPs; *n* = 105).

First, we found that the eight-week mindfulness intervention did not result in reductions in impulsivity as measured by either the BIS-11 or the go/no-go task, and did not result in any neurobiological changes, compared to either active or wait-list control groups. Importantly, this lack of change in impulsivity co-occurred with a significant increase in mindfulness, as reported by a previous study from our group on this sample and intervention^67^. This indicates that despite the MBSR intervention working as intended by increasing mindfulness, it was not effective for decreasing impulsivity.

Second, we found that while LTMs self-reported heightened capacity to maintain attention compared to MNPs, they did not display heightened capacity to withhold prepotent motor responses on the go/no-go task, and, surprisingly, self-reported higher scores on the motor and non-planning subscales of the BIS-11. Together with the intervention results, these findings suggest that neither short-term nor long-term mindfulness practice may be effective for reducing impulsivity derived from deficits in inhibitory motor control or planning capacity in otherwise healthy adults, but that, consistent with other literature, long-term meditative practice is associated with heightened attentional control.

We also found that LTMs had lower frontostriatal gray matter volume, heightened resting-state functional connectivity between the basal ganglia, midbrain, thalamus and cortex, and lower spontaneous eye-blink rate compared to MNPs. In our group’s previous study of the MNPs in this sample^17^, these neurobiological signatures were found to be associated with increased levels of impulsivity. In particular, less volume in medial orbitofrontal cortex and paracingulate gyrus was associated with higher BIS-11 motor impulsivity and non-planning impulsivity subscale scores. Here, LTMs were found to have less volume than MNPs in these same two areas, and consistently, reported higher BIS-11 motor impulsivity and non-planning impulsivity subscores. In addition, in the previous study, lower spontaneous eye-blink rate was also found to be associated with higher BIS-11 motor impulsivity subscale score; here, LTMs had lower spontaneous eye-blink rates than MNPs in conjunction with higher BIS-11 motor impulsivity subscale score. However, while in the previous study increased resting-state functional connectivity between the midbrain, striatum, thalamus and prefrontal cortex was associated with decreased motor inhibition on the go/no-go task, LTMs did not differ from MNPs on go/no-go task performance despite greater functional connectivity compared to MNPs between these regions. Importantly, none of these neurobiological metrics were significantly associated with lifetime practice hours in LTMs. This suggests that rather than being related to mindfulness meditation practice itself, the neurobiological differences in LTMs compared to MNPs may more likely be due to preexisting differences in the unique subset of the population that chooses to spend large amounts of time practicing mindfulness meditation. It is worth noting, though, that if the effects of meditative practice do not accumulate linearly or at the same pace in all LTMs, this could result in a lack of correlation between lifetime practice hours and the neurobiological metrics too.

A secondary finding of interest was that subjects in the active-control HEP group did experience a significant decrease in self-reported BIS-11 impulsivity after the intervention. We considered several explanations for why subjects in the HEP intervention, but not subjects in the MBSR intervention, experienced a reduction in impulsivity. First, our group’s previous study of this sample and intervention^67^ found that subjects in the HEP group experienced a significant increase in mindfulness relative to the passive-control WL group. However, this increase in mindfulness was not significantly different than that experienced by the MBSR group. The lack of correspondence between impulsivity and mindfulness changes in the MBSR group suggested that the HEP group’s decrease in impulsivity may be unrelated to their increase in mindfulness. We provided more direct support for this here by showing that changes over time in BIS-11 impulsivity were not correlated with changes over time in mindfulness in individuals in the HEP group. Second, we observed that HEP subjects completed significantly more home practice hours than MBSR subjects over the course of the intervention. However, the amount of home practice hours was not found to be significantly associated with the degree of change in BIS-11 scores over time - either collapsed across groups or within either the MBSR or HEP group. More home practice hours by HEP subjects therefore does not appear to explain why they experienced self-reported impulsivity reductions and MBSR subjects did not. Third, post-hoc analysis revealed a strong gender effect, such that males in the HEP group experienced a significantly greater reduction in self-reported impulsivity than females. This was related to the surprising finding that more home practice hours were actually associated with less impulsivity reduction in HEP females, whereas no effect was seen in HEP males. This gender effect was not observed in the MBSR group. However, since gender composition did not significantly differ between the MBSR and HEP groups, gender per se is also unlikely to account for the difference between groups in impulsivity reduction. Thus, the difference may be attributable to aspects of the HEP intervention’s programming itself that are distinct from aspects of the MBSR intervention’s programming. For instance, in place of sitting meditation, HEP has a nutrition education component that includes meal planning and diet tracking homework. It is possible that this type of programming, which promotes the implementation of daily behaviors related to deliberately planning out meals and inhibiting intake of unhealthy foods, gives participants a sense of improved self-control and reduced impulsivity.

Several methodological issues should be considered in interpreting the findings from this study. First, as alluded to earlier, the cross-sectional design of the LTM vs. MNP comparisons precludes determination of the extent to which group differences in impulsivity and neurobiology reflect effects of long-term meditation practice separate from effects of self-selection and pre-existing differences. Indeed, the absence of a general pattern of significant correlations between lifetime practice hours and the impulsivity or neurobiological metrics suggests that these findings might be primarily attributable to pre-existing differences. Individuals who self-select to spend substantial time meditating may be a unique group. Furthermore, it is possible that this unique group of individuals comprising the LTMs may interpret the BIS-11 self-report differently than the general population (MNPs) or may hold themselves to different standards. For instance, endorsing the item “I am more interested in the present than the future” would increase a subject’s non-planning impulsivity BIS score, yet this concept is at the core of mindfulness practice – being mindful of and attentive to the present moment. Mindfulness and impulsiveness both share a focus on the present^5^. However, whereas impulsivity arises from hasty and heedless reactions to present circumstances, mindfulness is defined by a heightened awareness of the present that is nonreactive and observant in nature. The BIS-11 may not be optimally designed to pick up on this subtle yet crucial distinction, and so scores for LTMs - and potentially for the MBSR group after the intervention - may be incorrectly inflated. To examine whether this had a meaningful effect on the current findings, we reanalyzed the BIS-11 data without this item, and found that its exclusion did not change any of the results (see Supplemental Analysis 2). Nonetheless, future studies examining impulsivity in long-term meditators may wish to use self-report measures other than the BIS-11 or use an array of behavioral tasks to measure different dimensions of impulsivity.

In interpreting the findings from the intervention, it is first important to note that the particular mindfulness meditation intervention used here, MBSR, is just one of a number of kinds of mindfulness meditation interventions. While MBSR may not be effective in reducing impulsivity in healthy adults, other studies may wish to examine the effects of alternative forms of mindfulness meditation on impulsivity. Future studies may also wish to investigate the efficacy of mindfulness interventions for reducing impulsivity in younger participants, such as teenagers or young adults. Furthermore, the results from this healthy sample do not necessarily suggest that use of this intervention in clinical populations with abnormally high levels of impulsivity would be ineffective.

Overall, the largely null findings from this study suggest that any effect of MBSR on impulsivity in healthy adults is negligible.

## Supplementary information

Supplementary Info

## Data Availability

The datasets generated during and/or analyzed during the current study are available from the corresponding author on reasonable request.
