# Supplementary material for: The Effect of Mindfulness Meditation on Impulsivity and its Neurobiological Correlates in Healthy Adults
Source: Sci Rep. 2019 Aug 19;9:11963. doi: 10.1038/s41598-019-47662-y (PMC6700173; doi:10.1038/s41598-019-47662-y)
Supplement: Supplementary file 1 — Supplementary Info [file 41598_2019_47662_MOESM1_ESM.docx]

**SUPPLEMENTAL MATERIALS:**

**The Effect of Mindfulness Meditation on Impulsivity and its Neurobiological Correlates in Healthy Adults**

Cole Korponay^1,4^ Ph.D., ckorponay@wisc.edu

Daniela Dentico^1,4,5^, MD, Ph.D., dentico@wisc.edu

Tammi R.A. Kral^2,4,5^, M.S., kral@wisc.edu

Martina Ly^4,5^, Ph.D., mly2@wisc.edu

Ayla Kruis^4,5,6^, MSc, a.kruis@uva.nl

Kaley Davis^4^, B.A., keedwards2@wisc.edu

Robin Goldman^4,5^, Ph.D., rigoldman@wisc.edu

Antoine Lutz^3,4,5^, Ph.D., antoine.lutz@inserm.fr

Richard J. Davidson^1,2,4,5^, Ph.D., rjdavids@wisc.edu

*^1^Department of Psychiatry, University of Wisconsin-Madison, Madison, Wisconsin, 53719, USA*

*^2^Department of Psychology, University of Wisconsin–Madison, Madison, Wisconsin, 53706, USA*

*^3^Lyon Neuroscience Research Center, Brain Dynamics and Cognition Team, INSERM U1028, CNRS UMR5292, Lyon 1 University, Lyon, France*

*^4^Center for Healthy Minds, University of Wisconsin–Madison, Madison, Wisconsin, 53703, USA*

*^5^Waisman Laboratory for Brain Imaging and Behavior, University of Wisconsin–Madison, Madison, Wisconsin, 53705, USA*

*^6^ University of Amsterdam, 1012 WX Amsterdam*, *Netherlands*

**Table S1. Relative and Absolute RMS means; Number of Frame-wise Displacements (FDs) > 0.2 mm**

FD > 0.2 mm (mean counts)

LTM: 15.7

MBSR T1: 10.9

MBSR T2: 25.8

HEP T1: 15.3
HEP T2: 19.2

WL T1: 15.6
WL T2: 21.1

Relative RMS (means)

LTM: 0.06

MBSR T1: 0.06

MBSR T2: 0.07

HEP T1: 0.06
HEP T2: 0.06*

WL T1: 0.06
WL T2: 0.07

Absolute RMS (means)

LTM: 0.28

MBSR T1: 0.11

MBSR T2: 0.46

HEP T1: 0.32
HEP T2: 0.34*

WL T1: 0.35
WL T2: 0.36

*Data from one HEP T2 subject excluded due to a single large motion spike (this spike was filtered out of any statistical analyses via motion censoring)

**Table S2. Subjects Included vs. Subjects Excluded from RSFC Analyses due to Motion**

|  |  |
| --- | --- |
|  | ***p^a^*** |
| *Age* | 0.95 |
| *Raven IQ* | 0.22 |
| *Household Income* | 0.99 |
| *Gender* | 0.55 |
| *Caucasian* | 0.48 |
| *Asian* | 0.39 |
| *American Indian or Alaska Native* | 0.10 |
| *Mixed* | 0.79 |
| *Did Not Report* | 0.46 |

**Table S3. Impulsivity Analyses with Outliers Excluded**

Go Trial Accuracy, LTM vs. MNP: F(1,126)=4.345, p=0.039, B=-3.32

No-Go Syllable Trial Accuracy, LTM vs. MNP: F(1,126)=0.013, p=0.910, B=-0.31

Post-error Slowdown, LTM vs. MNP: F(1,126)=2.772, p=0.098, B=24.72

Motor Impulsivity, group x time: F(2,98)=0.116, p=0.891

Go Trial Accuracy, group x time: F(2,96)=0.684, p=0.507

No-Go Repeat Trial Accuracy, group x time: F(2,97)=1.079, p=0.344

No-Go Syllable Trial Accuracy, group x time: F(2,96)=0.062, p=0.940

Post-Error Slowdown, group x time: F(2,98)=0.868, p=0.423

**Table S4. Means and Standard Deviations of BIS-11 Total Scores**

|  | **Mean** | **SD** |
| --- | --- | --- |
| **WL T1** | 59.8 | 7.75 |
| **WL T2** | 58.4 | 9.63 |
| **HEP T1** | 56.67 | 7.47 |
| **HEP T2** | 54.08 | 7.60 |
| **MBSR T1** | 60.94 | 8.61 |
| **MBSR T2** | 60.79 | 8.73 |
| **LTM** | 62.57 | 8.10 |

**Supplemental Analysis 1: Cortical Thickness in FreeSurfer**

To corroborate the null findings with regard to gray matter changes over the course of the intervention as measured by VMB in SPM, we conducted supplementary surface-based cortical thickness analyses in FreeSurfer. For preprocessing, images first underwent FreeSurfer's automated preprocessing procedure, which includes skull-stripping, registration, intensity normalization, Talairach transformation, tissue segmentation, and surface tessellation (Fischl and Dale, 2000). Images then underwent manual inspection and editing to ensure precise and accurate segmentation of gray and white matter. Consistent with the VBM findings, no significant differences were observed between groups in the change in cortical thickness from Time 1 to Time 2, with the exception of a MBSR vs. WL difference in postcentral gyrus, but this was driven by a change within the WL group.

**Supplemental Analysis 2: BIS-11 Non-Planning Impulsivity with Item 27 ("I am more interested in the present than the future) excluded**

Group x Time: F(2,99)=3.399, p=0.037

LTMs vs. MNPs: F(1,129)=3.958, p=0.049.

Both findings remain significant even after exclusion of Item 27.
